# Validation of an ovine lung parenchyma material model with pressures representing the breathing cycle for high-velocity impacts

**DOI:** 10.1007/s10237-026-02124-z

**Published:** 2026-09-27

**Authors:** Patricia K. Thomas, Eugene Ablordeppey, Grace K. Liverett, Philip J. Brown, F. Scott Gayzik

**Affiliations:** Department of Biomedical Engineering, Wake Forest School of Medicine, 575 N. Patterson Ave Suite 530, Winston-Salem, NC 27101 USA

**Keywords:** Finite element modeling, Lung biomechanics, Digital image correlation, Validation

## Abstract

Lung injury risk from high-velocity blunt impacts is thought to depend on lung pressure. Risk is typically assessed with finite element models (FEMs), but models do not typically account for lung pressure, nor do they account for lung viscoelasticity. The objective of this study is to translate and then validate a set of lung material models that consider both tissue viscoelasticity and pressure in silico*.* This was completed using one set of quasi-static simulations for translation and one set of dynamic simulations for validation to compare to experimental forces, where CORA was utilized. Digital image correlation (DIC) data of strain and displacement magnitude from the quasi-static experiments were also used to ensure proper material model translation. Three simulations were run for each loading case, where the only difference was the lung material properties to account for pressure (0, 4, and 10 cmH_2_O). The force–time responses for both simulations showed good agreement with experimental data with a range of 0.77–0.93 CORA scores for the dynamic loading, and a range of 0.84–0.97 CORA scores for the quasi-static simulations, with all simulations falling under “good” or “excellent” categories. The DIC-equivalent data for the quasi-static simulations were determined to be dependent on mesh size. Overall, the quasi-static simulations showed the material was translated into LS-Dyna well, and each of the lung material models were able to reproduce the global force–time responses well in the dynamic simulation set.

**Supplementary Information:**

The online version contains supplementary material available at https://doi.org/10.1007/s10237-026-02124-z.

## Introduction

35% of trauma-related deaths in the USA are thoracic trauma, where pulmonary contusion is the most common injury (Edgecombe and Angus 2018). Depending on the situation in which trauma occurs, up to 50% of blunt trauma cases result in pulmonary contusion injuries, but are known to range from 30 – 75% depending on age and the mechanism of injury (Rendeki and Molnár 2018; Choudhary et al. 2024). Pulmonary contusions lead to edema and inflammation, impeding gas exchange within the lung parenchyma, creating a ventilation–perfusion mismatch, which can be fatal (Choudhary et al. 2024). Both rib and sternal fractures are thought to be correlated with pulmonary contusions due to the polytraumatic nature of how they most often occur (Rendeki and Molnár 2018; Choudhary et al. 2024). Injury risk is thought to be dependent on internal pressures, such as those throughout breathing, particularly when high-velocity pressure waves are the cause of injury (Stuhmiller, 2012; Thomas et al. 2025a).

Pulmonary contusions and thoracic trauma are often studied in post-mortem human subjects (PMHS), animal subjects, and computational models. Computational models provide localized mechanical data points that go beyond what can typically be observed in conventional impact biomechanics studies of PMHS and animal subjects, including soft tissue parenchymal stresses, strains, and pressure data. Such data that involve instrumentation would likely disrupt tissue responses in experimental models. However, computational models are only as good as their inputs, e.g., their geometries and their material model definitions. For instance, lung tissue is known to be hyperelastic and viscoelastic, with some material changes due to the tissue pressure, however, many lung models in silico neglect the viscoelasticity of the tissue as well as the internal lung pressure (Thomas et al. 2025a; Zeng et al. 1987; Rater 2013; Hoppin et al. 1975; Vawter et al. 1979, 1978). In contrast, many ex vivo lung testing experiments noted creep and viscoelastic responses during tests (Thomas et al. 2025a; Zeng et al. 1987; Rater 2013; Hoppin et al. 1975; Vawter et al. 1979, 1978).

There are several large thoracic or whole-body finite element models (FEMs) available for use in simulating blunt impacts, such as those from motor vehicle crashes or high-rate non-penetrating blunt injury (NPBI) (Gepner et al. 2022; Caffrey et al. 2024; Gayzik et al. 2009; Davis and Gayzik 2016; Caffrey et al. 2024). Human body models such as Global Human Body Modeling Consortium (GHBMC), Total HUman Model for Safety (THUMS), and SAFER utilize more simplified material models due to the large size of the FEMs and the computational costs associated with the larger model (Rater 2013; Gayzik et al. 2009; Larsson 2023; Gepner et al. 2022; Davis and Gayzik 2016; Matsuda et al. 2023; Pipkorn, et al. 2021). SAFER is based on the THUMS model, where the THUMS model v7.1 utilizes an elastic fluid material model for lung parenchyma, which requires the density, elastic modulus, Poisson’s ratio, and bulk modulus (Matsuda et al. 2023; LS-DYNA, 2021). The SAFER v11 was updated to utilize a low-density foam model based on inversely calculated coefficients (Rater 2013)*.* This model doesn’t consider the viscoelasticity appropriately, as by definition, the shear modulus is negligible in Newtonian fluids. Other versions of THUMS have used a low-density foam, which does not account for internal lung pressures, nor does it account for viscoelasticity (Rater 2013; Matsuda et al. 2023). The lung material model used by GHBMC utilizes a simplified rubber material model based on the data from *Eaton *et al*.*, which also fails to account for tissue viscoelasticity (Eaton et al. 2021; Elemance, Global Human Body Models Consortium 2021). Another model, known as the finite element ovine thorax model (FE-OTM), which was developed for high-velocity NPBIs, utilizes a simplified rubber material model with the same data from *Eaton *et al*.* as GHBMC (Caffrey et al. 2024; Caffrey et al. 2024; Eaton et al. 2021). None of these models consider the viscoelasticity of the lung tissue, which may lessen the biofidelity of the behavior due to the lack of consideration for tissue relaxation during impacts. The lack of viscoelasticity therefore changes the resulting injury risk assessment, particularly in high-velocity blunt impacts.

While there are many studies that have characterized the material behavior of lung parenchyma, most do not account for the internal lung pressures, and only one to the authors’ knowledge has tested over a range of pressures (Thomas et al. 2025a; Eaton et al. 2021; Silva et al. 2009; Andrikakou et al. 2016). The objective of this study is to translate experimental results into LS-Dyna and then to validate multiple in silico material models representing three physiologically relevant lung pressures in LS-Dyna for high-velocity impacts based on work completed by *Thomas *et al*.* and *Andrikakou *et al. (Thomas et al. 2025a; Andrikakou et al. 2016*).*

## Methods

### Specimen collection

All ovine samples were collected following USDA standards from a local abattoir immediately after sacrifice (N = 10). Lung samples were separated laterally and inflated to 20 cmH_2_O using a continuous positive airway pressure device (CPAP) (Airsense 10, Resmed, San Diego, CA, USA) during transport to the laboratory until testing. The CPAP had a reported measurement uncertainty of ± 0.15 cmH_2_O according to the manufacturer. CPAP tubing was connected to the secondary bronchiole and secured with a custom adapter that press-fit the connector to the tissue. No further separation was done for the remainder of the testing, and the same airway connection was maintained. A non-damaging set of tests was completed as described by *Thomas *et al*.* prior to dynamic testing (Thomas et al. 2025a). Samples were periodically sprayed with saline to maintain hydration. All dynamic impact tests were completed within 8 h of sacrifice. Samples were therefore fresh, non-frozen tissue.

### Experimental eCCI dynamic impacts

The two largest and most caudal lobes were used for the high-velocity impact tests. Some samples were discarded due to tissue damage, as they were unable to inflate. One pressure, either 0, 4, or 10 cmH_2_O was selected for each impact, where one impact was completed on each lobe (N = 15). Each lobe was impacted once at a velocity of 4 m/s to a depth of 10 mm from the surface of the tissue. These pressures were the pressures utilized by *Thomas *et al*.,* and represent 20, 50, and 80% of the lung vital capacity, respectively based on a reported pressure percent vital capacity curve from *Parent *et al*.* (Parent 2015).

**Fig. 1:**
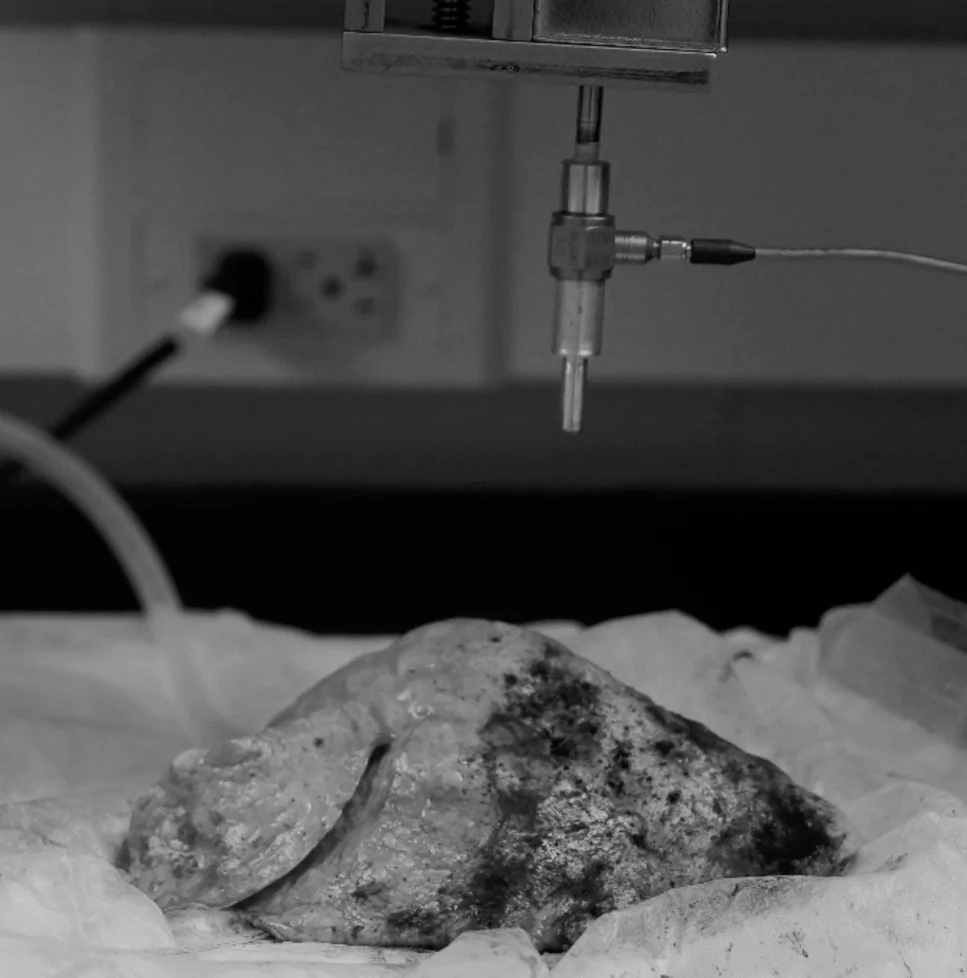
eCCI and lung set-up prior to high-velocity impact

High-velocity impact tests were completed using an electric cortical contusion impactor (eCCI) (Custom Design & Fabrication Inc, Sandston VA) instrumented with a piezoelectric load cell (208C02, PCB Piezotronics, Depew NY) with broadband resolution of 0.004 N-rms sampled at 48 kHz. Before each impact, the impactor displacement was zeroed at the surface of the lung while ensuring good contact. The target for impact was chosen to minimize edge effects and chosen to occur on a thicker portion of the lobe. The anvil (aluminum, diameter = 10 mm) struck the samples at 4 m/s to a 10-mm depth. The anvil, load cell, and adapter were rigidly attached to eCCI, as shown in Fig. 1. The resulting force–time data traces were filtered using a SAE J211 CFC 1000 filter, and then inertially compensated due to the large accelerations during the impact. Data traces were then shortened to solely include the impact from impactor contact to peak depth for in silico validation.

Statistical analysis was completed on the resulting energies and peak forces from the impacts using JMP version 17.0 (SAS Institute, Cary, NC). A mixed model was utilized, where the animal subject was a random effect, with the lobe nested. The pressure for the impact was an additional variable (α = 0.05).

### Simulations

#### Simulation geometry and material inputs

All simulations were run in LS-Dyna (ANSYS, Canonsburg PA) using solver version 9.3.1 on a standard 8 core local machine. Two sets of simulations were run—one simulation set was a quasi-static spherical material test matching the experiments completed by *Thomas *et al*.* The other simulation set matched the dynamic testing that was previously described, for a total of 6 simulations across both simulation sets. Using both sets of simulations ensures that the material is properly translated from experiments into the finite element environment and then validated within a dynamic environment for high-velocity applications. Both simulations utilized a lung geometry that matched a representative lung from experimental testing.

**Fig. 2:**
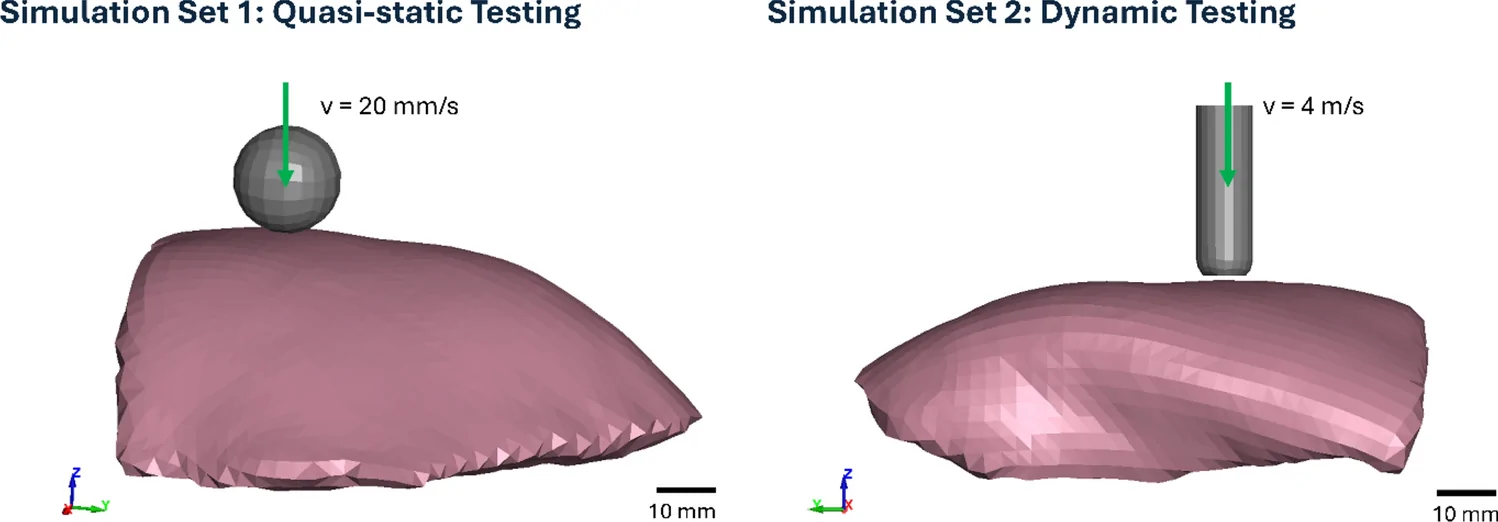
Simulation set-ups for each test. (Left) The quasi-static indentation set-up. (Right) Dynamic testing set-up. On both, the green arrow indicates the direction of loading and is labeled with the loading velocity

After all experimental testing was completed, the intact lung lobe was scanned using the Peel 3 3D scanner (Peel 3D, Levis, Qc, Canada). This resulted in a point cloud, which was meshed using tetrahedral elements (~ 14 k elements, average edge length of 4.3 mm, and Jacobian values greater than 0.3) using Geomagic (3D Systems, Rock Hill, SC) and ANSA (Beta CAE Systems, Farmington Hills, MI). Element size was selected to match existing models, such as the FE-OTM in literature, in order to make this validation applicable for use in whole-body models (Caffrey et al. 2024). A convergence study was completed, where the selected element size is larger than the converged size would be, as a compromise between mesh sensitivity and applicability was needed. Element quality was verified in Hypermesh (Altair Development, Troy, MI). The final lung mesh is visible in Fig. 2. Within the lung part, only lung parenchyma is modeled. Due to the material model incorporating pressure within the coefficients as the model varies by pressure, and the experimental set-ups include positive pressure within each lung, minimal air is expected to be lost in either of the ex vivo and in the in silico set-ups.

Three different hyper viscoelastic material models were used in LS-Dyna. The hyperelastic portion of the models utilized an Ogden formulation, and the viscoelastic portion used a Prony series formulation. The hyperelastic inputs included the stress–strain relationship of lung tissue reported by *Andrikakou *et al*.* for all three versions of the material model (Andrikakou et al. 2016). The Ogden model was used as opposed to a purely viscoelastic model to ensure model stability during dynamic tests. Ogden formulations are known to be more stable, able to handle large deformations, and can have material descriptions such as viscoelasticity added to better predict tissue behavior (LS-DYNA, 2021). However, it is worth noting that Ogden models assume isotropy and require a more thorough evaluation of the material model due to different loading parameters, hence a higher loading rate and a test set-up that is more representative of the high-velocity NPBI environment (LS-DYNA, 2021).

As for the viscoelasticity portion of the model, viscoelasticity data from *Thomas *et al*.* was utilized (Thomas et al. 2025a). Other lung material models have been found throughout literature, and have similar magnitudes of constants such as shear modulus, but due to the viscoelasticity model available within LS-Dyna as part of the Ogden material, the model from *Thomas *et al*.* was utilized (Thomas et al. 2025a; Sattari and Eskandari 2020; Badrou et al. 2025; Polio et al. 2018). Three different sets of viscoelastic parameters were used to represent different internal lung pressures. The pressures represented were 0, 4, and 10 cmH_2_O, resulting in three simulations per loading protocol, where a Prony series was utilized (Eq. 1, Table 1) (Andrikakou et al. 2016). Note that the shear coefficient units are in gigapascals (GPa) for unit consistency (kg-mm-ms). Poisson’s ratio was defined for the lung tissue, where a value of 0.42 was used for all three pressures (Polio et al. 2018). While the ratio is commonly reported to be around 0.42, values as low as 0.22 have also been reported, which would in turn affect the parenchyma behavior (Al‐Mayah et al. 2009; Freed et al. 2012; Butler et al. 1986; Concha et al. 2018). Varying mass densities were utilized, as the mass density changes as the air pressure increases due to the increase in air volume within the lung parenchyma. Mass density was estimated based on sheep lung mass (559 g), associated air volume, which was based on the vital capacity (60 mL/kg) at each pressure for a 35-kg sheep, and the estimated tissue volume (Zhou and Zhang 2018; Huchon et al. 1986; Marshall and Mayer 2007). The tissue volume was estimated based on a literature mass density value of 0.25 g/cm^3^, where the tissue volume was found to be 1816 mL (Zhou and Zhang 2018). Different mass densities were considered important, as the mass density directly affects the speed of sound.

**Table 1:** Lung material properties used in silico for *MAT_Ogden_Rubber (Thomas et al. 2025a)

Pressure (cmH_2_O)	ρ (kg/mm^3^)	C_0_ (GPa)	C_1_ (GPa)	β_1_ (1/ms)	C_2_ (GPa)	β_2_ (1/ms)	C_3_ (GPa)	β_3_ (1/ms)
0	2.50e−07	3.74e−6	3.96e−54	2.22	9.45e−7	2.40e−5	1.01e−6	3.82e−4
4	1.95e−07	3.30e−6	5.05e−54	2.22	1.04e−6	3.30e−5	1.32e−6	5.40e−4
10	1.59e−07	2.65e−6	5.58e−54	2.22	1.08e−6	2.80e−5	1.47e−6	4.89e−4

1 $$G\left(t\right)= {C}_{0}+ \sum {C}_{k}{e}^{-{\beta}_{k}t}$$

#### eCCI simulation set-up and boundary conditions

The first simulation modeled the dynamic eCCI test that was described previously using an explicit solver in LS-Dyna with three simulations, where each represents a different pressure. The surface of the lung tissue in contact with the support table was constrained using a set of nodes constrained in all three dimensions to prevent movement of the base of the tissue throughout the impact. This constraint mirrored observations of the testing. The rigid impactor was built using 525 hexahedral elements, matching the geometry of the tip of the eCCI with aluminum material properties (diameter = 10 mm), as seen in Table 2. The tip was then translated close to the tissue surface while ensuring a perpendicular impact angle. The tip was prescribed to impact the tissue at 4 m/s to a depth of 10 mm, and the simulation was terminated the instant the anvil leading edge reached peak depth. The contact force was filtered using a low pass J211 SAE 1000 filter and the resulting force–time trace was analyzed with an object evaluation technique known as CORrelation and Analysis (CORA) (PDB, Gaimersheim, Germany) to validate the model.

**Table 2:** Impactor material properties for each simulation

Simulation	Material	Test Type	Material Constants
Indentation	Delrin*	Quasi-static (Thomas et al. 2025a)	E = 3.1 GPa, ρ = 1.41e−6 kg/mm^3^, ν = 0.37
eCCI	Aluminum**	Dynamic	E = 68.9 GPa, ρ = 1.3e−6 kg/mm^3^, ν = 0.34, K = 0.21, N = 0.13

*Rigid material model,

**Power law plasticity material model

#### Indentation simulation set-up and boundary conditions

The second simulation modeled the quasi-static material test completed by *Thomas *et al*.* using an implicit–explicit switching solver in LS-Dyna*.* Both are utilized, as an explicit solver was needed to minimize instabilities during initial contact, whereas an implicit solver was needed due to the long duration and quasi-static nature of the simulation. The original spherical indentation experiments utilized a low friction spherical indenter made of Delrin, and a high friction surface for the sample to sit on to prevent lateral movement (Thomas et al. 2025a). The Delrin indenter impacted the tissue at a rate of 20 mm/s perpendicular to the surface of the sample to a depth of 10 mm and maintained that depth for 120 s (Thomas et al. 2025a). Three simulations were run in this simulation set, where each represented a different pressure.

A rigid shell sphere with 150 elements was meshed and translated to be close to the lung surface to ensure perpendicular contact with the tissue in a representative location similar to experiments, as described by *Thomas *et al. (Thomas et al. 2025a). Delrin material properties per the manufacturer were used in a rigid material, as seen in Table 2, which details the impactor material properties for each set-up. The bottom nodes were constrained using a set of nodes constrained in all three dimensions to prevent movement of the base of the tissue to match experimental conditions. The indenter loaded the tissue at a rate of 20 mm/s to a depth of 10 mm, where peak depth was maintained for 40 s to collect force–time histories for comparison to experimental data traces. A shorter time was chosen than what was done experimentally due to the computational expense of a long simulation, even with the use of an implicit solver. 40 s was determined to be the time where at least 90% of the relaxation had occurred across all three pressure states during experiments, so it was selected for the maximum simulated time (Thomas et al. 2025a).

The contact force between the impactor and the lung was then filtered using a low pass J211 SAE 1000 filter, where the data was used to verify proper translation in CORA. To compare to the resulting digital image correlation (DIC) data from *Thomas *et al*.*, nodal displacement magnitudes and elemental strains at three radial distances of 5, 10, 15, 20, and 30 mm using consistent nodes and elements were investigated and compared to the results from *Thomas *et al*.* for additional verification of translation.

#### CORA validation

CORA was used to quantitatively assess the biofidelity of the lung through the contact forces for each simulation set. All default settings within CORA v.3.51 were used, along with the rating system described by *Lalwala *et al., where scores between 0.65 and 0.85 are good, and above 0.85 is considered excellent (Correia et al. 2021; Lalwala et al. 2023; Östh et al. 2014).

## Results

### Experimental eCCI dynamic impact results

A total of 6 lung samples were tested at 0 cmH_2_O, 4 samples were tested at 4 cmH_2_O, and 5 samples were tested at 10 cmH_2_O. The average peak velocity was 4.00 ± 0.02 m/s, and the average peak displacement was 10.11 ± 0.02 mm.

**Fig. 3:**
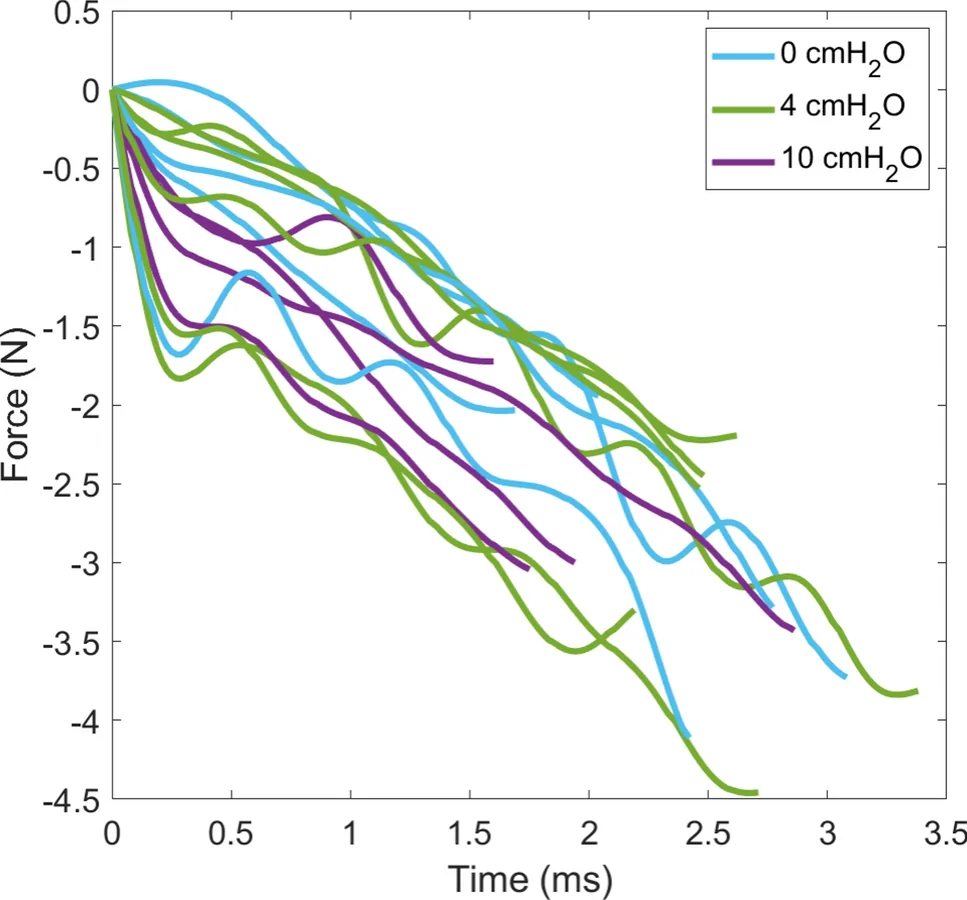
Resulting eCCI impact traces colored based on pressure

The peak force and total energies for the impacts were not significant by pressure (p > 0.4). There were no visible striations based on pressure, as shown in Fig. 3. No visible tissue damage was left from the impact after an impact despite the dynamic nature of the test. However, it is worth noting that other behaviors, such as wave propagation or strain fields were not investigated and may be different between pressures.

### eCCI simulation validation

**Fig. 4:**
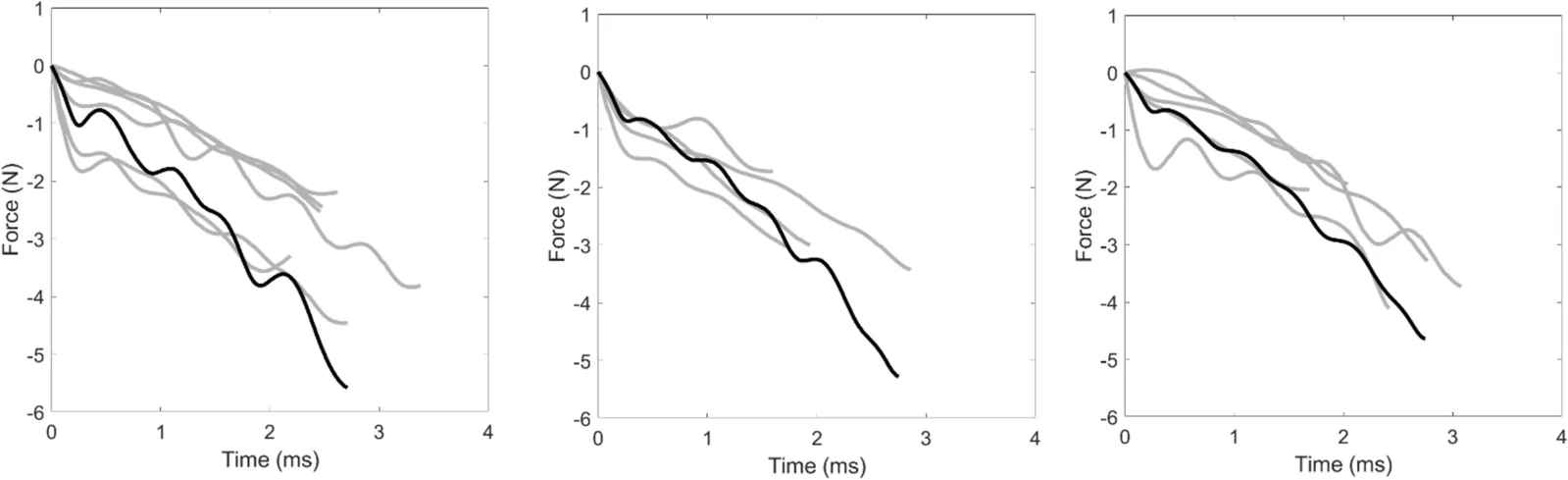
Resulting eCCI simulation data traces (black) for (Left) 0 cmH_2_O, (Middle) 4 cmH_2_O, and (Right) 10 cmH_2_O compared to experimental data traces (gray)

The eCCI data traces and simulation data are shown for all tested pressures in Fig. 4. The experimental data traces for the 0 cmH_2_O group are less grouped than 4 and 10 cmH_2_O, leading to more visual misalignment, which is partially due to the stratification in the experimental data set.

All dynamic simulations showed strong agreement with the experimental data. Quantitatively, the 0 cmH_2_O group had a lower CORA score than the other two groups, as seen in Table 3, likely due to the visible split in the data traces for the 0 cmH_2_O group (Fig. 4). In Table 3, we see the 0 cmH_2_O group has a “good” score, whereas the 4 and 10 cmH_2_O groups have “excellent” scores (Lalwala et al. 2023). All three material models successfully reproduced the global force–time response for the dynamic impacts.

**Table 3:** Resulting CORA scores for eCCI Impacts

Pressure (cmH_2_O)	CORA Score
0	0.77
4	0.93
10	0.86

### Indentation simulation translation

**Fig. 5:**
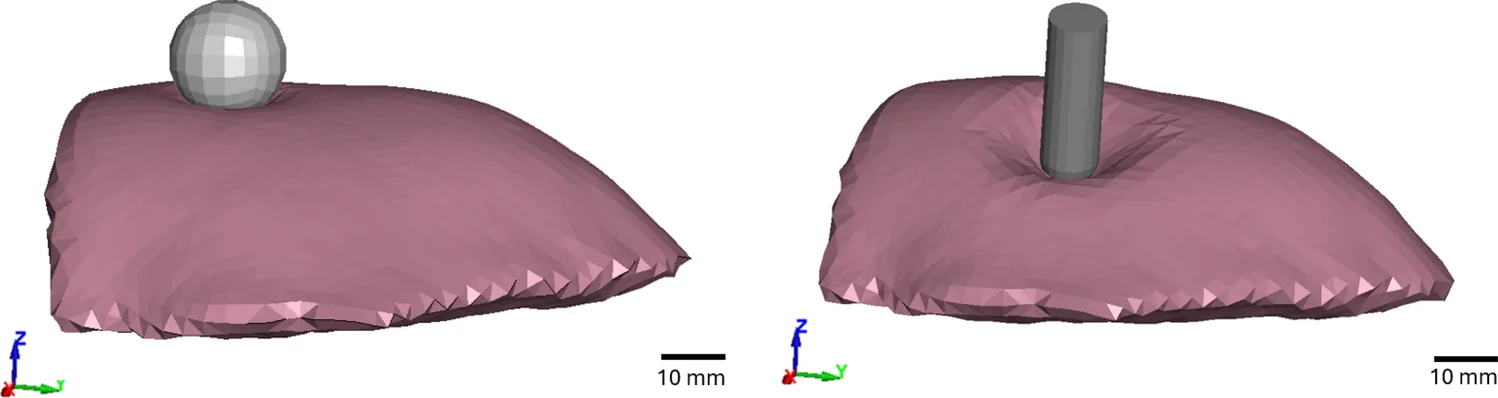
(Left) Spherical indentation simulation and (Right) dynamic simulation at peak depth

Throughout each indentation simulation, the tissue maintained stability and visually matched the spherical indentation experiments, showing that the material model translated well into LS-Dyna. A frame of the simulation post peak depth is shown in Fig. 5, where the spherical indenter is at peak displacement.

Overall, the indentation simulations resulted in “good” and “excellent” matches when compared to experimental data using CORA (Table 4), where both 4 and 10 cmH_2_O were considered “excellent” matches (Correia et al. 2021; Lalwala et al. 2023; Östh et al. 2014).

**Table 4:** Resulting CORA scores for indentation simulations

Pressure (cmH_2_O)	CORA Score
0	0.84
4	0.95
10	0.97

**Fig. 6:**
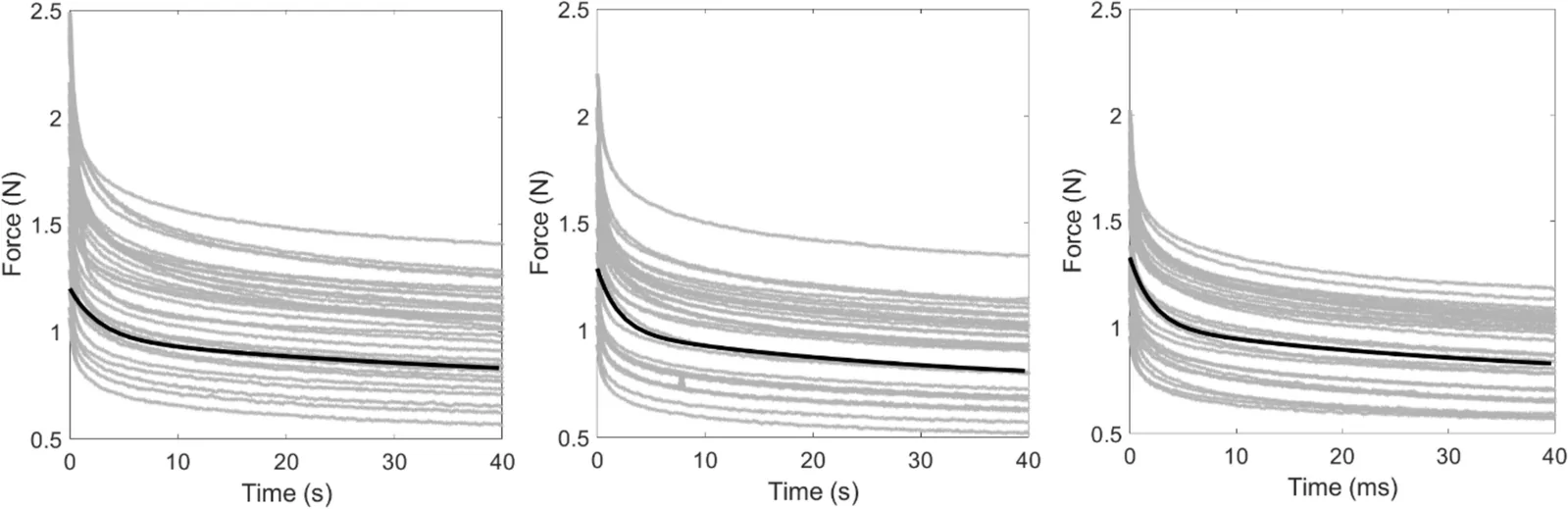
Spherical indentation simulation results (solid black line) compared to experimental results (gray lines) from (Thomas et al. 2025a) for (Left) 0 cmH_2_O, (Middle) 4 cmH_2_O, and (Right) 10 cmH_2_O

As shown in Fig. 6, the simulation outputs visibly line up with experimental data traces that are in gray for each pressure. Therefore, the three material models are considered to have been properly translated into the finite element environment from experimental results.

**Fig. 7:**
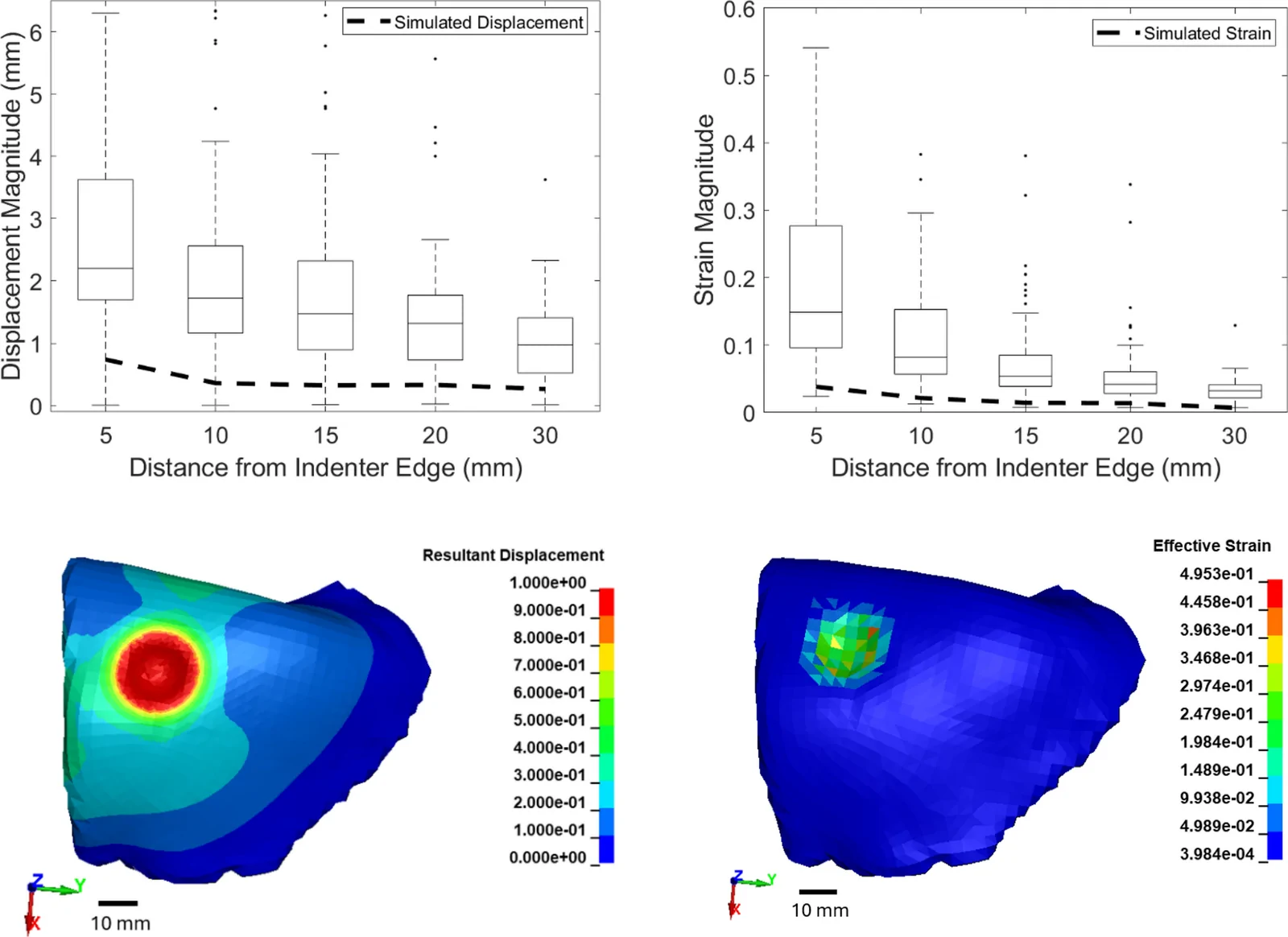
Resulting DIC comparisons for (Top, Left) displacement magnitude on a linear axis of lung tissue and (Top, Right) strain magnitude on a linear axis at various distances from indenter edge, where the dashed lines are simulation data, and the solid lines with standard deviation bars are the experimental values. (Bottom, Left) Example of fringe plot with resultant displacement range from aerial view. (Bottom, Right) Fringe plot with strain magnitude

The DIC strain field from *Thomas *et al*.* had no differences by pressure. Similarly, the displacement and strain magnitudes in silico were also not different by pressure. This is likely due to the nature of the test, as it was a displacement-controlled test. The mean displacements and strains for each simulation, regardless of pressure, were identical. With this in mind, Fig. 7 shows all the data consolidated by pressure, both in graphical format and a fringe plot format. Regardless of how far the measurement was from the indenter edge, the simulation values are lower than those in experiments for both displacement and strain magnitudes. These differences are potentially due to mesh size sensitivities, where significantly smaller element sizes would result in increased sensitivity, and therefore be closer to experimental results. However, the magnitudes of both the strain and displacement magnitudes are of similar scale to experiment and within experimental bounds.

## Discussion

This study showed that the experimental dynamic impact forces and total energies were not considered statistically different across the three pressures tested. Previous experimental literature was successfully translated into LS-Dyna, as shown by the quasi-static simulations. The translated material models were then validated using a dynamic simulation set at each lung pressure from *Thomas *et al*.*, allowing for the model to be used in NPBI applications (Thomas et al. 2025a)*.*

While there were no statistical differences between the three pressures experimentally, additional behaviors not specifically investigated may be different. With the pressure differences, the ratio of air to tissue leads to different speeds of sound throughout the lung parenchyma (Rice 1983). Particularly in high-velocity impacts, this is important to account for, as strain fields would in turn be affected, ultimately affecting injury outcomes. For example, one organ level threshold found to be indicative of injury is strain x strain rate, which is directly impacted by the speed of sound (Gayzik et al. 2007). Due to the validated lung material models being intended for use in larger models like the FE-OTM and GHBMC for lung injury risk analysis, it is important to account for additional behaviors outside of force and energy, such as strain fields.

**Fig. 8:**
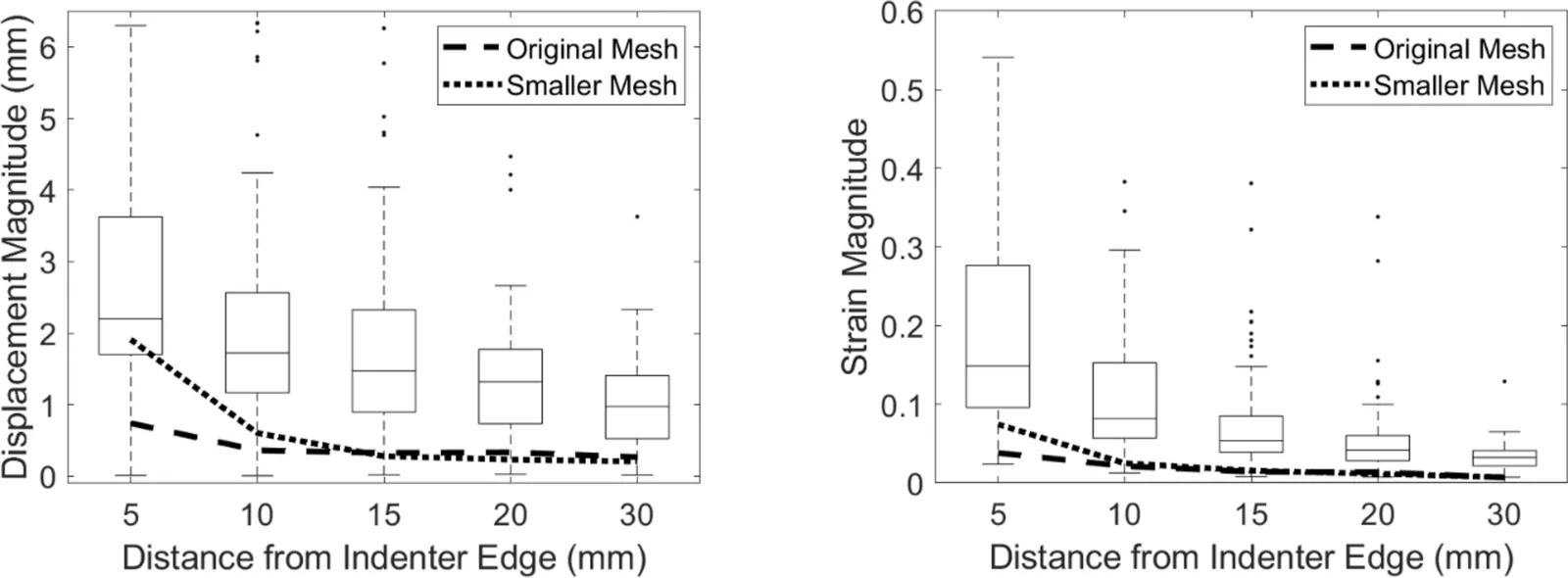
Smaller mesh size DIC analysis results for (left) displacement magnitude, and (right) strain magnitude compared against the experimental data (in boxplots) and original simulation results

The strains and displacement magnitudes were analyzed similar to the methods reported by *Thomas *et al*.*, where consistent nodes and elements were utilized in silico. While there was no visible difference experimentally nor computationally by pressure looking at the strain fields, one is anticipated and may be observed through other parameters that were not investigated due to viscoelastic behavior differences by pressure (Thomas et al. 2025b). There are limitations with this analysis, as mesh size will affect the resulting strains and displacements. With the chosen element size larger than a fully converged element size, an additional simulation was run using the 0 cmH_2_O material with lung elements split four ways in LS-PrePost for an edge length of 1 mm, for a total of 57,904 elements. Analysis of the resulting simulation showed changes in both the displacement and strain values in silico, as shown in Fig. 8. The assumed Poisson’s ratio of 0.42 may also affect the strain fields, particularly if lowered to a value closer to 0.3.

The smaller element size affected the values closer to the indenter the most (Fig. 8). The changes for both strain and displacement are due to mesh size, as the larger elements resulted in lower strains and displacements than the simulation with smaller elements. Both simulations underestimated the strains compared to experiment, however, the smaller elements are now closer to the median of most of the measurements from experiments, where all the displacements were small. Element edge length closer in size to the resolution of the original DIC experimental work completed would likely result in computational data closer to the experimental data. The stress–strain curve from *Andrikakou *et al*.* is limited by the test set-up, as the tissue used was excised and unable to be pressurized, affecting outputs such as strain fields based on wave propagation within the tissue (Andrikakou et al. 2016; Rice 1983). It is important to note that for pulmonary contusion applications, the injury is typically localized to the point of impact, showing that this model is still applicable for modeling blunt thoracic trauma and studying pulmonary contusions but may still under-predict the strains in vivo when using 1st principal injury strain metrics (Gayzik 2007; Caffrey 2026, Danelson 2011, Danelson 2015).

Since the Ogden material model is limited as it requires a stress–strain curve input, it is important to note that other material models could have been utilized in validating the lung material model validated in this paper. For other applications, particularly those that have slower loading and/or smaller elemental strains, a viscoelastic-specific material model could be utilized that would not require the stress–strain curve. In the case of instability, contact schemes that prevent full compression may be used to assist in maintaining positive element volumes. However, due to the objective to validate this material model for a range of applications, *MAT_OGDEN_RUBBER was the material model selected in LS-Dyna for this work, as it maintains element positive volumes better than other material models within this solver. Other material models in LS-Dyna used in previous include *MAT_SIMPLIFIED_RUBBER and *MAT_LUNG_TISSUE, which have limitations surrounding viscoelasticity, biofidelity, and/or stability in high-rate loading environments (Gepner et al. 2022; Caffrey et al. 2024; Matsuda et al. 2023; Gayzik et al. 2007). Another consideration would be the Poisson’s ratio value—the behavior and strains would change with a lower ratio. It should also be noted that the Poisson’s ratio of the bulk material likely changes with air pressure, where lower pressures likely have lower ratios, affecting the parenchyma behavior (Hoppin et al. 1975; Denny and Schroter 2006). Additionally, it is worth noting that this model did not consider the airway, which will affect overall organ behavior and needs to be considered for future applications. During indentation and dynamic ex vivo experiments, the airway was avoided due to potential edge effects and changes in behavior.

A limitation of the model used within this study includes the lack of damage. Other more detailed and custom constitutive models, such as models by *Clayton *et al*.*, incorporate damage within the model (Clayton et al. 2021). However, with models as complicated as these, specific element types, such as a hex element, are required, along with the specific model code, making it more difficult for the end user. This model has the benefit of being able to be easily used by users due to the lack of requirements surrounding element types and lack of custom code. While the model presented in this study does not have damage, future work can include the addition of damage through the use of readily available *MAT_ADD_DAMAGE_DIEM or *MAT_ADD_DAMAGE_GISSMO cards within LS-Dyna (LS-DYNA, 2021).

The applications of this model include wound healing and improved injury risk curve development. Mechanical strains have been indicated to cause microinjury in disease states such as idiopathic pulmonary fibrosis (IPF), in traumatic scenarios such as motor vehicle crashes and high-rate blunt impacts (Gayzik et al. 2007; Thomas et al. 2025c; Felder et al. 2019; Savla and Waters 1998). One work indicated that strains above 5% may be correlated to IPF (Felder et al. 2019). While the intended application of this work is for high-rate loading to evaluate trauma, the model may still be used within quasi-static applications such as ventilation to better understand risk of injury and/or disease states such as IPF (Savla and Waters 1998).

Additional work should be done to understand the mesh sensitivity of the strains within lung tissue, which will be necessary for studies investigating injury risk of lung parenchyma due to the strain-based injury metrics of the tissue (Gayzik et al. 2007). Strain analysis using this model with a coarse mesh will likely underestimate the strains in vivo. However, the resulting material can be applied to a wide range of finite element models that undergo various impacts or loading conditions at different stages in the breathing cycle. Ovine subjects are commonly used throughout literature as lung injury models due to similar lung parenchyma anatomy compared to humans. Specifically, alveolar diameters are similar in size between human and ovine species, allowing for a more direct translation to human applications for a range of lung trauma scenarios (Lum and Mitzner 1987). These include, but are not limited to, full human body finite element models, animal models, or thoracic models that are subjected to a large range of loading conditions such as car crashes, high-velocity impacts, high-velocity pressure waves, or sport impacts with higher biofidelity and better predictive qualities than previously used lung material models.

## Supplementary Information

Below is the link to the electronic supplementary material.

Supplementary file1 (DOCX 693 KB)

## Data Availability

Data will be available upon request.

## Abbreviations

DIC: Digital image correlation
eCCI: Electric cortical contusion impactor
FEM: Finite element model
FE-OTM: Finite element ovine thorax model
GHBMC: Global Human Body Modeling Consortium
IPF: Idiopathic pulmonary fibrosis
NPBI: Non-penetrative blunt injury
PMHS: Post-mortem human subjects
SAFER: Side impact family of dummies with enhanced restraints
THUMS: Total HUman Model for Safety
