# Supplementary material for: Validation of an ovine lung parenchyma material model with pressures representing the breathing cycle for high-velocity impacts
Source: Biomech Model Mechanobiol. 2026 Sep 27;25(5):114. doi: 10.1007/s10237-026-02124-z (PMC13616860; doi:10.1007/s10237-026-02124-z)
Supplement: Supplementary file 1 [file 10237_2026_2124_MOESM1_ESM.docx]

## Supplemental

### Mesh Convergence for Dynamic Simulations

A mesh convergence study was conducted to select a mesh size for validation work using the dynamic eCCI test described in the methods. Four different mesh sizes were tested – 1-, 5-, 10-, and 20-mm edge lengths, and the peak force was compared between mesh sizes (Figure 9). As the edge length decreased, the run time increased, where the 1-mm edge length case took 1 hour and 42 minutes to complete. Due to both computational power limitations and mesh convergence study results, the 5 mm edge length was the selected mesh size for this study (Table 5).

| Figure 9: Resulting mesh convergence study values, plotting peak force of eCCI impacts over the range of mesh edge lengths | | |
| --- | --- | --- |
| Table 5: Mesh size convergence values with resulting peak force and computational time | | |
| **Mesh Size (mm)** | **Peak Force (N)** | **Computational Time** |
| 1 | 4.21 | 1 hour 42 minutes |
| 5 | 5.19 | 2 minutes |
| 10 | 6.94 | 41 seconds |
| 20 | 27.7 | 37 seconds |

Peak Force (N)

| Mesh Size (mm) | Resultant force |
| --- | --- |
| 20 | 27.7 |
| 10 | 6.94 |
| 5 | 5.19 |
| 1 | 4.21 |

The 1 mm mesh size for 4 mmHg pressure was run to completion to determine the effect on the consistency with experimental data. The CORA score using default settings for the 1 mm mesh size was 0.71, which is still considered “good”, but the simulated data trace for the 1 mm mesh size was softer than the simulated data trace for 5 mm mesh size (Figure 10). The 5 mm mesh size better represents lung parenchyma behavior due to a CORA score closer to 1 (0.93) than the 1 mm mesh size (0.71).

Figure 10: CORA traces for 4 cmH2O material model with a 1-mm mesh size

### Poisson’s Ratio Sensitivity

An additional sensitivity study was completed due to the large range of Poisson’s ratio values in literature, where values range from 0.22 to 0.45. All simulations were identical, using the 5 mm mesh size and 4 cmH_2_O material model, where the only variable was the Poisson’s ratio. The study matrix with the resulting peak forces and CORA scores are seen in Table 5, where a total of 5 simulations were run, including the ratio reported in the main study (Simulation #4 in Table 5). With the simulation results in mind, it should be noted that variations in the Poisson’s ratio will affect the resulting forces and mildly affect the biofidelity of the model. All CORA was completed in version 3.51 with the default settings.

Table 5: Simulation matrix for Poisson’s ratio sensitivity analysis

| **Simulation #** | **Poisson’s Ratio** | **Peak Force (N)** | **CORA score** |
| --- | --- | --- | --- |
| 1 | 0.22 | 4.92 | 0.95 |
| 2 | 0.30 | 4.98 | 0.96 |
| 3 | 0.35 | 5.04 | 0.96 |
| 4 | 0.42 | 5.19 | 0.93 |
| 5 | 0.45 | 5.51 | 0.92 |

As shown in Table 5 and in Figure 11, the peak force increases with an increased Poisson’s ratio. Due to the increased incompressibility as the ratio increases, the material behaves stiffer, increasing that peak force. Figure 11 also shows increased oscillations with lower Poisson’s ratios. The CORA score does not large change, as all scores are considered “excellent” [36]. While the ideal ratio may be closer to 0.35, due to the larger volume of studies utilizing values ranging from 0.4 to 0.5, the ratio of 0.42 was used for the main study [28, 30, 50, 51].

Figure 11: Force–time traces for each Poisson’s ratio simulation

### Quasi-static DIC Mesh Convergence

A brief mesh convergence study was conducted to compare how the mesh size affects the strain and displacement magnitudes. Larger element sizes were not considered due to how poorly they perform in previous mesh convergence studies (see the “Mesh Convergence for Dynamic Simulations” section). A total of 3 simulations were run, with mesh sizes of 0.5, 1, and 5 mm. The resulting peak displacement and strain are show in Figure 12. The strain magnitude converges more quickly than the displacement magnitude between mesh sizes 0.5 to 1 mm, however, both metrics are converging at just under 1 mm mesh size.

Displacement Magnitude (mm)

| Mesh Size (mm) | In Silico Mesh Convergence |
| --- | --- |
| 5 | 0.739 |
| 1 | 1.91 |
| 0.5 | 1.98 |

| Mesh Size (mm) | Median Experimental Displacement Magnitude |
| --- | --- |
| 5.5 | 2.2 |
| 5 | 2.2 |
| 1 | 2.2 |
| 0.5 | 2.2 |
| 0 | 2.2 |

Strain Magnitude

| Mesh Size (mm) | In Silico Mesh Convergence |
| --- | --- |
| 5 | 0.0381 |
| 1 | 0.0746 |
| 0.5 | 0.0755 |

| Mesh Size (mm) | Median Experimental Strain Magnitude |
| --- | --- |
| 5.5 | 0.1487 |
| 5 | 0.1487 |
| 1 | 0.1487 |
| 0.5 | 0.1487 |
| 0 | 0.1487 |

Figure 12: Mesh convergence for local displacement (top) and strain (bottom) magnitudes, 5 mm from indenter edge.

### CORA Analysis and Figures

All CORA parameters used for analysis used the default CORA v3.51 settings. The corridors are shown in Figure 10. End times for evaluation of each pressure were determined by the shortest experimental data trace, hence different end times for evaluation.

Figure 11: CORA corridors for each pressure tested: (top) 0 cmH_2_O, (middle) 4 cmH_2_O, and (bottom) 10 cmH_2_O. All corridors were calculated using default CORA settings.
